# Insertion of the human sodium iodide symporter to facilitate deep tissue imaging does not alter oncolytic or replication capability of a novel vaccinia virus

**DOI:** 10.1186/1479-5876-9-36

**Published:** 2011-03-31

**Authors:** Dana Haddad, Nanhai G Chen, Qian Zhang, Chun-Hao Chen, Yong A Yu, Lorena Gonzalez, Susanne G Carpenter, Joshua Carson, Joyce Au, Arjun Mittra, Mithat Gonen, Pat B Zanzonico, Yuman Fong, Aladar A Szalay

**Affiliations:** 1 Department of Biochemistry, University of Wuerzburg, Wuerzburg, D-97074, Germany; 2 Department of Surgery, Memorial Sloan-Kettering Cancer Center, New York, NY 10065, USA; 3 Genelux Corporation, San Diego Science Center, San Diego, CA 92109, USA; 4 Departments of Medical Physics and Radiology, Memorial Sloan-Kettering Cancer Center, New York, NY 10065, USA; 5 Department of Radiation Oncology, University of California San Diego, San Diego, CA 92093, USA

## Abstract

**Introduction:**

Oncolytic viruses show promise for treating cancer. However, to assess therapeutic efficacy and potential toxicity, a noninvasive imaging modality is needed. This study aimed to determine if insertion of the human sodium iodide symporter (*hNIS*) cDNA as a marker for non-invasive imaging of virotherapy alters the replication and oncolytic capability of a novel vaccinia virus, GLV-1h153.

**Methods:**

GLV-1h153 was modified from parental vaccinia virus GLV-1h68 to carry *hNIS *via homologous recombination. GLV-1h153 was tested against human pancreatic cancer cell line PANC-1 for replication via viral plaque assays and flow cytometry. Expression and transportation of *hNIS *in infected cells was evaluated using Westernblot and immunofluorescence. Intracellular uptake of radioiodide was assessed using radiouptake assays. Viral cytotoxicity and tumor regression of treated PANC-1tumor xenografts in nude mice was also determined. Finally, tumor radiouptake in xenografts was assessed via positron emission tomography (PET) utilizing carrier-free ^124^I radiotracer.

**Results:**

GLV-1h153 infected, replicated within, and killed PANC-1 cells as efficiently as GLV-1h68. GLV-1h153 provided dose-dependent levels of *hNIS *expression in infected cells. Immunofluorescence detected transport of the protein to the cell membrane prior to cell lysis, enhancing hNIS-specific radiouptake (P < 0.001). *In vivo*, GLV-1h153 was as safe and effective as GLV-1h68 in regressing pancreatic cancer xenografts (P < 0.001). Finally, intratumoral injection of GLV-1h153 facilitated imaging of virus replication in tumors via ^124^I-PET.

**Conclusion:**

Insertion of the *hNIS *gene does not hinder replication or oncolytic capability of GLV-1h153, rendering this novel virus a promising new candidate for the noninvasive imaging and tracking of oncolytic viral therapy.

## Introduction

Oncolytic viral therapies have shown such success in preclinical testing as a novel cancer treatment modality that several phase I and II trials are already underway. Oncolytic vaccinia virus (VACV) strains have been of particular interest due to several advantages. VACV's large 192-kb genome enables a large amount of foreign DNA to be incorporated without reducing the replication efficiency of the virus, which has been shown not to be the case with some other viruses such as adenoviruses [1]. It has fast and efficient replication, and cytoplasmic replication of the virus lessens the chance of recombination or integration of viral DNA into cells. Perhaps most importantly, its safety profile after its use as a live vaccine in the World Health Organization's smallpox vaccination program makes it particularly attractive as an oncolytic agent and gene vector [2].

Currently, biopsy is the gold standard for monitoring the therapeutic effects of viral oncolysis [3-5]. This may be feasible in preclinical or early clinical trials, however, a noninvasive method facilitating ongoing monitoring of therapy is needed for human studies. The tracking of viral delivery could give clinicians the ability to correlate efficacy and therapy and monitor potential viral toxicity. Furthermore, a more sensitive and specific diagnostic technique to detect tumor origin and, more importantly, presence of metastases may be possible [3].

Here, we report on the construction and testing of a VACV carrying the human sodium iodide symporter (hNIS) as a marker gene for non-invasive tracking of virus by imaging. This virus was derived from VACV GLV-1h68, which has already been shown to be a simultaneously diagnostic and therapeutic agent in several human tumor models including breast tumors [6], mesothelioma [7], canine breast tumors [8], pancreatic cancers [9], anaplastic thyroid cancers [10,11], melanoma [12], and squamous cell carcinoma [13].

## Materials and methods

### Virus and cell culture

African green monkey kidney fibroblast CV-1 cells and human pancreatic ductal carcinoma PANC-1 cells were purchased from American Type Culture Collection (ATCC) (Manassas, VA) and were grown in Dulbecco's modified Eagle's medium (DMEM) supplemented with 1% antibiotic-antimycotic solution (Mediatech, Inc., Herndon, VA) and 10% fetal bovine serum (FBS) (Mediatech, Inc.) at 37°C under 5% CO_2_. Rat thyroid PCCL3 cells were a kind gift from the lab of Dr. James Fagin at MSKCC and were maintained in Coon's modified medium (Sigma, St. Louis, MO), 5% calf serum, 2 mM glutamine, 1% penicillin/streptomycin, 10 mM NaHCO3, and 6H hormone (1 mU/ml bovine TSH, 10 ug/ml bovine insulin, 10 nM hydrocortisone, 5 ug/ml transferrin, 10 ng/ml somatostatin, and 2 ng/ml L-glycyl-histidyl-lysine) at 37°C under 5% CO_2_. GLV-1h68 was derived from VACV LIVP, as described previously [6].

### Construction of hNIS transfer vector

The *hNIS *cDNA was amplified by polymerase chain reaction (PCR) using human cDNA clone TC124097 (SLC5A5) from OriGene as the template with primers hNIS-5 (5'-GTCGAC(Sal I) CACCATGGAGGCCGTGGAGACCGG-3') and hNIS-3 (5'-TTAATTAA(Pac I) TCAGAGGTTTGTCTCCTGCTGGTCTCGA-3'). The PCR product was gel purified, and cloned into the pCR-Blunt II-TOPO vector using Zero Blunt TOPO PCR Cloning Kit (Invitrogen, Carlsbad, California). The resulting construct pCRII-hNIS-1 was sequenced, and found to contain an extra 33-bp segment in the middle of the coding sequence, representing an alternative splicing product for hNIS. To remove this extra 33-bp segment, two additional primers were designed to flank the segment, and used in the next set of PCR. In the next round of reactions, hNIS-5 paired with hNIS-a3 (5'-GAGGCATGTACTGGTCTGGGGCAGAGATGC-3'), and hNIS-a5 (5'-CCCAGACCAGTACATGCCTCTGCTGGTGCTG-3') paired with hNIS-3 were used in separate PCRs, both with pCRII-hNIS-1 as the template. The respective PCR products were then mixed and used as the templates in one reaction with hNIS-5 and hNIS-3 as the primer pair. The final PCR product was again cloned into the pCR-Blunt II-TOPO vector as pCRII-hNISa-2, confirmed by sequencing to be identical to the SLC5A5 sequence in GenBank (accession number NM_000453). The hNIS cDNA was then released from pCRII-hNIS-1 with Sal I and Pac I, and subcloned into HA-SE-RLN-7 with the same cuts by replacing RLN cDNA. The resulting construct HA-SE-hNIS-1 were confirmed by sequencing and used for insertion of PE-hNIS into the HA locus of GLV-1h68.

### Generation of hNIS-expressing VACV

CV-1 cells were infected with GLV-1h68 at a multiplicity of infection (MOI) of 0.1 for 1 hour, then transfected using Fugene (Roche, Indianapolis, IN) with the hNIS transfer vector. Two days post infection, infected/transfected cells were harvested and the recombinant viruses selected and plaque purified as described previously [14]. The genotype of hNIS-expressing VACV GLV-1h153 was verified by PCR and sequencing. Also, expression of GFP and β-galactosidase was confirmed by fluorescence microscopy and 5-bromo-4-chloro-3-indolyl-β-D-galactopyranoside (X-gal, Stratagene, La Jolla, CA), respectively, and lack of expression of *gusA *was confirmed by 5-bromo-4-chloro-3-indolyl-β-D-glucuronic acid (X-GlcA, Research Product International Corp., Mt. Prospect, IL).

### Viral growth curves

PANC-1 cells were seeded onto 6-well plates at 5 × 10^5 ^cells per well. After 24 hours in culture, cells were infected with either GLV-1h153 or GLV-1h68 at an MOI of 0.01 or 1.0. Cells were incubated at 37°C for 1 hour with brief agitation every 30 minutes to allow infection to occur. The infection medium was then removed, and cells were incubated in fresh growth medium until cell harvest at 1, 24, 48, and 72 hours post infection. Viral particles from the infected cells were released by 3 freeze-thaw cycles, and the titers determined as (PFU/10^6^) in duplicate by plaque assay in CV-1 cell monolayers.

### Flow cytometry

Cells were seeded on 6-well plates at 5 × 10^5 ^cells per well. Wells were then infected at MOIs of 0, 0.01, and 1.0, and cells then harvested at 6, 12, 24, 48, 72, and 96 hours postinfection by trypsinizing and washing with phosphate-buffered saline (PBS). For the second experiments, cells were seeded on 6-well plates at 5 × 10^5 ^cells per well. Wells were then infected at MOIs of 0, 0.01, 0.1, 0.5, 1.0, 2.0, and 5, and were harvested in the same manner at 24 hours after infection. GFP expression was analyzed via a Becton-Dickinson FACScan Plus cytometer (Becton-Dickinson, San Jose, CA). Analysis was performed using CellQuest software (Becton-Dickinson).

### hNIS mRNA analysis via microarray

To evaluate the level of hNIS mRNA production in infected cells, cells were plated at 5 × 10^5 ^cells per well and infected with GLV-1h153 at an MOI of 5.0. Six and 24 hours postinfection, 3 samples of each time point were harvested and lysis performed directly using RNeasy mini kit protocol (Qiagen Inc., Valencia, CA). The mRNA samples were measured by spectrophotometer for proof of purity and hybridized to HG-U133A cDNA microarray chips (Affymetrix Inc, Santa Clara, CA) by the genomic core laboratory at Memorial Sloan-Kettering Cancer Center (MSKCC). The chip images were scanned and processed to CEL files using the standard GCOS analysis suite (Affymetrix Inc). The CEL files were then normalized and processed to signal intensities using the gcRMA algorithm from the Bioconductor library for the R statistical programming system. All subsequent analysis was done on the log (base 2) transformed data. To find differentially expressed genes a moderated t-test was used as implemented in the Bioconductor LIMMA package. To control for multiple testing the False Discovery Rate (FDR) method was used with a cutoff of 0.05.

### hNIS protein analysis via Western blot

To confirm whether the hNIS protein was being expressed in infected cells, cells were plated at 5 × 10^5 ^per well and infected with GLV-1h153 at various MOIs of virus, harvested at 24 hours, and suspended with SDS-PAGE and 0.5-m DDT reagent. After sonication, 30 ug of the protein samples were loaded on 10% Bis-Tris-HCl buffered polyacrylamide gels using the Bio-rad system (Bio-rad laboratories, San Francisco, CA). Following gel electrophoresis for 1 hour, proteins were transferred to nitrocellulose membranes using electroblotting. Membranes were then preincubated for 1 hour in 5% low fat dried milk in TBS-T (20 mm Tris, 137 mm NaCl, and 0.1% Tween-20) to block nonspecific binding sites. Membranes were incubated with a purified mouse antibody against hNIS at 1:100 dilution (Abcam Inc., Cambridge, MA) and incubated for 12 hour at +4°C. After washing with TBS-T, secondary antibody (horseradish peroxidase-conjugated goat antimouse IgG (Santa Cruz, Santa Cruz, California) was applied for 1 hour at room temperature at a 1:5,000 dilution. Peroxidase-bound protein bands were visualized using enhanced chemiluminescence Western blotting detection reagents (Amersham, Arlington Heights, IL) at room temperature for approximately 1 minute and using Kodak BIOMAX MR films for exposure. Normal human thyroid lysate was used as a positive control, and cells treated with GLV-1h68 and PBS were used as negative controls.

### Immunofluorescence

PANC-1 cells grown in a 12-well plate at 1 × 10^6 ^were mock-infected with GLV-1h68 or infected with GLV-1h153 at an MOI of 1.0. Twenty-four hours after infection the cells were fixed with 3.7% paraformaldehyde, permeabilized with methanol, blocked with PBS containing BSA, and incubated with a mouse anti-hNIS monoclonal antibody (Abcam Inc., Cambridge, MA) at a dilution of 1:100, followed by incubation with a secondary red fluorochrome-conjugated goat antimouse antibody (Invitrogen) at a dilution of 1:100. Pictures were taken using a Nikon inverted fluorescence microscope.

### *In vitro *radiouptake assay

Radio-uptake in cells infected with GLV-1h153 was compared to rat thyroid cell line PCCL3 endogenously expressing NIS and to cells infected with parental virus GLV-1h68. Cells were plated at 5 × 10^5 ^per well in 6-well plates. Twenty-four hours after infection with MOIs of 0.01, 0.10, and 1.0, cells were treated with 0.5 μCi of either carrier-free ^131^I or ^131^I with 1 mM of sodium perchlorate (NaClO4), a competitive inhibitor of hNIS, for a 60-minute incubation period. Media was supplemented with 10 μM of sodium iodide (NaI). Iodide uptake was terminated by removing the medium and washing cells twice with PBS. Finally, cells were solubilized in lysis buffer for residual radioactivity, and the cell pellet-to-medium activity ratio (cpm/g of pellet versus cpm/mL of medium) calculated from the radioactivity measurements assayed in a Packard γ-counter (Perkin Elmer, Waltham, MA). Results were expressed as change in uptake relative to negative uninfected control. All samples were done in triplicate.

### *In vitro *cytotoxicity assay

PANC-1 pancreatic cancer cells were plated at 2 × 10^4 ^per well in 6-well plates. After incubation for 6 hours, cells were infected with GLV-1h153 or GLV-1h68 at MOIs of 1.00, 0.10, 0.01, and 0 (control wells). Viral cytotoxicity was measured on day 1 and every second day thereafter by lactate dehydrogenase (LDH) release assay. Results are expressed as the percentage of surviving cells as compared to uninfected control.

### *In vivo *tumor therapy studies and systemic toxicity

All mice were cared for and maintained in accordance with animal welfare regulations under an approved protocol by the Institutional Animal Care and Use Committee at the San Diego Science Center, San Diego, California. PANC-1 xenografts were developed in 6- to 8-week-old male nude mice (NCI:Hsd:Athymic Nude-*Foxn1*nu, Harlan) by implanting 2 × 10^6 ^PANC-1 cells in PBS subcutaneously in the left hindleg. Tumor growth was recorded once a week in 3 dimensions using a digital caliper and reported in mm^3 ^using the formula (length × width × [height-5]). When tumors reached 100-300 mm^3^, mice were injected intratumorally (IT) or intravenously (IV) via the tail vein with a single dose of 2 × 10^6 ^PFUs of GLV-1h153 or GLV-1h68 in 100 μL PBS. Animals were observed daily for any sign of toxicity, and body weight checked weekly.

### Radiopharmaceuticals

^124^I and ^131^I were obtained from MSKCC's radiopharmacy. The maximum specific activities for the ^124^I and ^131^I compounds were ~140 μCi/mouse and ~0.5 μCi/well, respectively.

### *In vivo *PET imaging

All animal studies were performed in compliance with all applicable policies, procedures, and regulatory requirements of the Institutional Animal Care and Use Committee, the Research Animal Resource Center of MSKCC, and the National Institutes of Health "Guide for the Care and Use of Laboratory Animals." Three groups of 2-3 animals bearing subcutaneous PANC-1 xenografts on the left hindleg measuring were injected intratumorally with 2 × 10^7 ^PFU GLV-1h153 (3 mice), 2 × 10^7 ^PFU GLV-1h68 (2 mice), or PBS (2 mice). Two days after viral injection, 140 μCi of ^124^I was administered via the tail vein. One hour after radiotracer administration, 3-dimensional list-mode data were acquired using an energy window of 350 to 700 keV, and a coincidence timing window of 6 nanoseconds. Imaging was performed using a Focus 120 microPET dedicated small animal PET scanner (Concorde Microsystems Inc, Knoxville, TN). These data were then sorted into 2-dimensional histograms by Fourier rebinning. The image data were corrected for (a) nonuniformity of scanner response using a uniform cylinder source-based normalization, (b) dead time count losses using a single-count rate-based global correction, (c) physical decay to the time of injection, and (d) the ^124^I branching ratio. The count rates in the reconstructed images were converted to activity concentration (%ID/g) using a system calibration factor (MBq/mL per cps/voxel) derived from imaging of a mouse-size phantom filled with a uniform aqueous solution of ^18^F. Image analysis was performed using ASIPro (Siemens Pre-clinical Solutions, Knoxville, TN).

### Statistical analysis

The GraphPad Prism 5.0 program (GraphPad Software, San Diego, CA) was used for data handling and analysis. The significance of differences between the 3 therapy groups (untreated, GLV-1h153, GLV-1h68) was determined via two-way ANOVA with Bonferroni correction. P values were generated for radiouptake assay comparisons using Dunnett's test [15]. P < 0.05 was considered significant.

## Results

### Construction of the hNIS transfer vector

**Figure 1:**
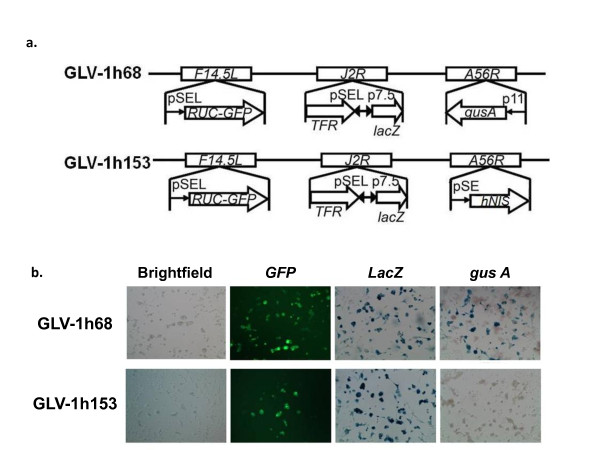
**GLV-1h153 construct**. a. GLV-1h153 was derived from GLV-1h68 by replacing the *gus A *expression cassette at the *A56R *locus with the *hNIS *expression cassette through *in vivo *homologous recombination. Both viruses contain RUC-GFP and *lacZ *expression cassettes at the *F14.5L *and *J2R *loci, respectively. PE, PE/L, P11, and P7.5 are VACV synthetic early, synthetic early/late, 11K, and 7.5K promoters, respectively. TFR is human transferrin receptor inserted in the reverse orientation with respect to the promoter PE/L.b. Confirmation of *GFP*, *LacZ*, and lack of *gus A *marker gene expression in GLV-1h153 infected CV-1 cells. While the *gus A *gene cassette is expressed in cells infected with parent virus GLV-1h68, this has been replaced by the *hNIS *gene cassette in GLV-1h153, leading to loss of *gus A *expression.

The GLV-1h153 construct used in this study was derived from GLV-1h68 by replacing the β-glucuronidase (*gusA*) expression cassette at the *A56R *locus with the hNIS expression cassette (SE-hNIS) containing the hNIS cDNA under the control of the VACV synthetic early promoter, by homologous recombination in infected cells. The genotype of GLV-1h153 (Figure 1a) was verified by PCR and sequencing, and the lack of β-glucuronidase expression was confirmed by X-GLcA staining (Figure 1b).

### GLV-1h153 replicated efficiently in PANC-1 cells

**Figure 2:**
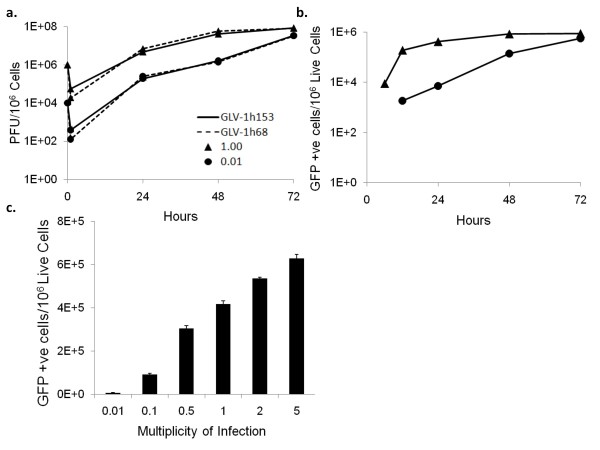
**Viral proliferation assay of GLV-1h153-in PANC-1 cells**. a. PANC-1 cells were grown in 6-well plates and infected with GLV-1h153 or GLV-1h68 at an MOI of 0.01 and 1.0. Three wells of each virus were harvested at 1, 24, 48, and 72 hours postinfection. GLV-1h153 replicated in a similar manner to GLV-1h68, with a 4-log increase in viral load at an MOI of 0.01 by 72 hours, reaching similar levels as that in cells infected with an MOI of 1.0. This demonstrates that GLV-1h153 is able to replicate efficiently within PANC-1 cells *in vitro *as well as parental virus GLV-1h68. b. GFP expression was quantified via flow cytometry in PANC-1 cells infected with GLV-1h153 at MOIs of 1.0 and 0.01 and was shown to be MOI dependent. GFP expression mimicked the viral replication growth curve, with GFP expression in the MOI 0.01 infected cells reaching similar levels as the MOI of 1.0 by 72 hours after infection. c. GFP expression was quantified via flow cytometry in PANC-1 cells infected with an MOI of 0.01, 0.1, 0.5, 1.0 2.0, and 5.0 at 24 hours after infection, and was shown to be MOI-dependent.

To evaluate the replication efficiency and effect of hNIS protein expression on VACV replication, PANC-1 cells were infected with either GLV-1h153 or its parental virus, GLV-1h68, at MOIs of 0.01 and 1.0, and the infected cells harvested at 1, 24, 48, and 72 hours post infection. The viral titers at each time point were determined in CV-1 cells using standard plaque assays. Both GLV-1h153 and GLV-1h68 replicated in PANC-1 cells at similar levels, indicating that the hNIS protein did not hinder viral replication within cells. GLV-1h153 yielded a 4-log, or 10,000-fold, increase of viral load with an MOI of 0.01 only 72 hours after infection. Within this time, viral load with an MOI of 0.01 reached the same levels as infection with an MOI of 1.0, again indicating efficient replication (Figure 2a).

### GLV-1h153 replication was assessed via flow cytometric detection of GFP

GFP expression in cells infected with either GLV-1h68 or GLV-1h153 was quantified using flow analysis, and was shown to be both time and MOI dependent. Adjusting for background, GFP expression mimicked the viral replication growth curve, with GFP expression in cells infected at an MOI of 0.01 reaching similar levels to an MOI of 1.0 by 72 hours (Figure 2b). Further, >70% of live cells expressed GFP at an MOI of 5.0 at 24hrs postinfection (Figure 2c).

### Production of hNIS mRNA and protein in infected cells was shown via microarray analysis and Western blot

**Figure 3:**
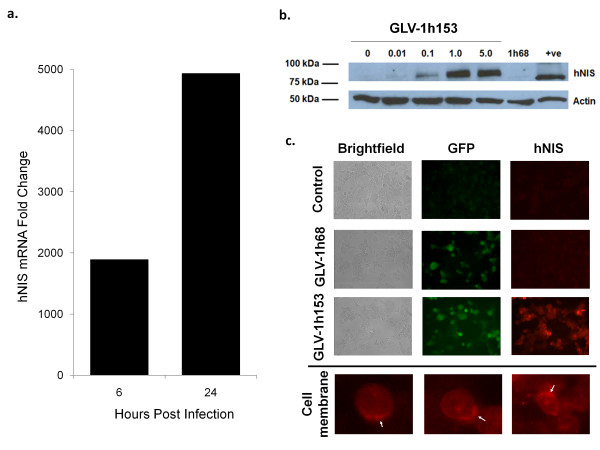
**Assessment of hNIS expression in GLV-1h153-infected PANC-1 cells**. a. Microarray analysis of cells infected with an MOI of 5.0 of GLV-1h153 yielded an almost 2000-fold increase by 6 hours and an almost 5000-fold increase by 24 hours in hNIS mRNA production as compared to noninfected control. b. PANC-1 cells were either mock infected or infected with GLV-1h68 at an MOI of 1.0 or infected with GLV-1h153 at an MOI of 1.0 or 5.0 for 24 hours. The hNIS protein was detected by Western blot analysis using monoclonal anti-hNIS antibody. Only GLV-1h153-infected cells expressed the hNIS protein, but cells either mock infected or infected with GLV-1h68 did not. The molecular weight marker bands (in kiloDaltons) are shown on the left. c. PANC-1 cells were mock infected or infected with GLV-1h68 or GLV-1h153 at a MOI of 1.0 for 24 hours. The hNIS protein was detected by immunofluorescence microscopy using monoclonal anti-hNIS antibody, which recognizes the intracellular domain of the protein. Mock- or GLV-1h68-infected cells (as demonstrated by GFP expression) did not express the hNIS protein, whereas the hNIS protein on the cell membrane of PANC-1 cells infected with GLV-1h153 was readily detectable.

To confirm production of hNIS mRNA by GLV-1h153-infected PANC-1 cells, cells were infected at an MOI of 5.0 and mRNA isolated for analysis with Affymetrix chips. mRNA in cells had an almost 2000-fold increase by only 6 hours after infection, and a >5000-fold change by 24 hours (P < 0.05) (Figure 3a). To show hNIS protein expression by GLV-1h153, PANC-1 cells were mock infected or infected with GLV-1h153 or parental virus GLV-1h68 at MOIs of 0.1, 1.0, and 5.0 and harvested 24 hours after infection. Production of the hNIS protein was successfully detected by Western blot between 75 and 100 KiloDalton, with an increasing concentration of protein at higher MOIs (Figure 3b). The difference in molecular weight of hNIS between the positive control and infected cells is likely due to the different levels of glycosylation, as noted by several other groups [16,17].

### The hNIS protein was localized at the cell membrane of PANC-1 cells

To determine whether the hNIS protein expressed by GLV-1h153 was successfully transported and inserted on the cell membrane, PANC-1 cells were infected with GLV-1h153 and fixed with 3.7% paraformaldehyde. The hNIS protein was visualized using a monoclonal anti-hNIS antibody that recognizes the intracellular domain of the protein. As shown in Figure 3c, mock- or GLV-1h68-infected cells (as demonstrated by GFP expression) did not show hNIS protein expression, whereas the hNIS protein in cells infected with GLV-1h153 was readily detectable by immunofluorescence microscopy, and appears to be localized at the cell membrane.

### GLV-1h153-infected PANC-1 cells showed enhanced uptake of carrier-free radioiodide

**Figure 4:**
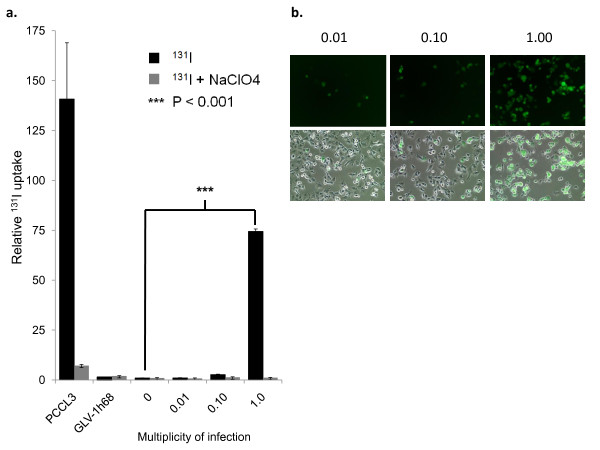
**Assessment of *in vitro *^131^I radiouptake of GLV-1h153-infected PANC-1 cells**. a. PANC-1 cells were infected with an MOI of 0, 0.01, 0.1, and 1.0 of GLV-1h153 and MOI of 1.0 of GLV-1h68. PCCL3 was used as a positive control. Twenty-four hours after infection, there is a >70-fold enhanced radiouptake at an MOI of 1.0 as compared to an MOI of 0 in GLV-1h153, and radiouptake is shown to be MOI dependent and hNIS specific (as shown with blocking with competitive inhibitor of hNIS, NaClO4). b. Maximum radiouptake with an MOI of 1.0 24 hrs after infection corresponded to maximum GFP expression.

To establish that the hNIS symporter was functional, cells were mock infected or infected at an MOI of 1.0 with GLV-1h153 and GLV-1h68, then treated with ^131^I at various times after infection. Normal rat thyroid cell line PCCL3 was used as a positive control. PANC-1 cells infected with GLV-1h153 showed a >70-fold increased radiouptake compared with mock-infected control at 24 hours post infection (P < 0.0001) despite similar cell protein levels, compared to 2.67 and 1.01-fold increased radiouptake with MOIs of 0.1 and 0.01, respectively (Figure 4a). This increased uptake correlated with peak GFP expression (Figure 4b). Moreover, when cells were treated with NaClO4, a competitive inhibitor of hNIS, radiouptake decreased in GLV-1h153-treated cells, from a 70- to a 1.14-fold difference at an MOI of 1.0, indicating hNIS-specific radiouptake. Radiouptake in cells infected with GLV-1h153 was compared to rat thyroid cell line endogenously expressing NIS (PCCL3), and to cells infected with parental virus GLV-1h68 or mock infected. Cells were plated at 5 × 10^5 ^cells per well in 6-well plates. Twenty-four hours after infection, cells were treated with 0.5 μCi of either carrier free ^131^I or ^131^I with 1 mM of sodium perchlorate (NaClO4), a competitive inhibitor of hNIS for a 60-minute incubation period. Media was supplemented with 10 μM of sodium iodide (NaI). Iodide uptake was terminated by removing the medium and washing cells twice with PBS. Finally, cells were solubilized in lysis buffer for residual radioactivity, and the cell pellet-to-medium activity ratio (cpm/g of pellet/cpm/mL of medium) calculated from the radioactivity measurements assayed in a Packard γ-counter (Perkin Elmer, Waltham, MA). Results are expressed as change in uptake relative to negative uninfected control. All samples were done in triplicate.

### GLV-1h153 was cytolytic against PANC-1 cells *in vitro*

**Figure 5:**
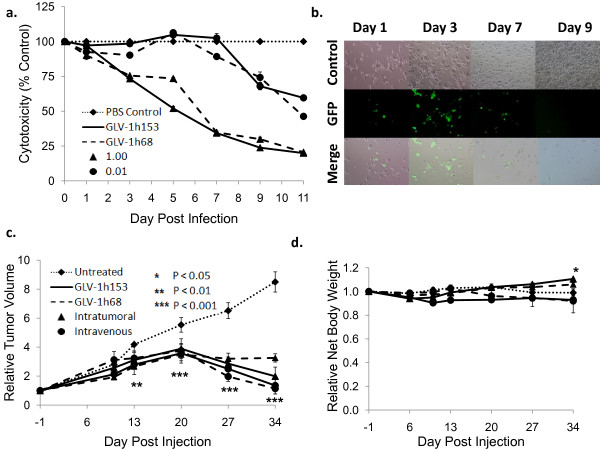
**GLV-1h153 infection and killing in cell culture and *in vivo***. a. PANC-1 cells were infected by various GLV-1h153at MOIs of 0.01, 0.1, and 1.0. Cell viability was determined via lactate dehydrogenase assays, and was set at 100% before infection. GLV-1h153 infected and was cytotxic at various MOIs, with less than 20% survival of cells as compared to control at an MOI of 1.0 by day 9. The values are the mean of triplicate samples, and bars indicate SD. b. GFP expression is shown to be time-dependent, with abundant GFP expression by day 3. Phase overlay pictures shows gradual cell death and thus decline of GFP expression by day 7. Closer examination of infected cells reveals loss of normal morphology and cell progressive cell detachment. c. 2 × 10^6 ^PFUs of GLV-1h153 or GLV-1h68, or PBS were injected IVly or ITly into nude mice bearing s.c. PANC-1 tumors on the hindleg (~100 mm^3^). GLV-1h153 was able to regress pancreatic tumor xenograft both ITly and IVly starting at day 13. The values are a mean of 4-5 mice, with bars indicating SEM. d. GLV-1h153 infection of pancreatic tumor xenografts did not have adverse effects on body weight at 5 weeks post injection, with the IT group even gaining weight compared to control.

To investigate whether expression of hNIS would affect cytolytic activity of VACV in cell cultures, PANC-1 cells were infected with GLV-1h68 or GLV-1h153 at MOIs of 0.01and 1.0. Viral cytotoxicity was measured every other day for 11 days. The survival curves for GLV-1h68 and GLV-1h153 were almost identical at both MOIs, indicating that the cells infected by either of the virus strains were dying at similar levels in a time- and dose-dependent fashion (Figure 5a). By day 11, More than 60% cell kill was achieved with an MOI of 1.0 as compared to control.

### GLV-1h153 was safe and effective at regressing PANC-1 tumor xenografts *in vivo*

To establish cytolytic effects of GLV-1h153 *in vivo*, mice bearing PANC-1 xenograft tumors on hindleg were infected intratumorally ( ITly) or intravenously (IVly) with GLV-1h153 or GLV-1h168, or mock treated with PBS. While the growth of tumors treated with PBS continued to grow, GLV-1h153-treated tumors occurred in three distinct phases: growth, inhibition, and regression (Figure 5c). The mean relative size of tumors treated with GLV-1h153 was significantly smaller than untreated control tumors, with differences beginning as early as day 13 (P < 0.01), and continuing till day 34 after virus or PBS control administration (P < 0.001). By day 34, there was an over 4-fold difference between control and IV tumor volumes, and an over 6-fold difference in the IT group. Furthermore, there were no significant adverse effects seen with regard to body weight, with the IT group even gaining weight as compared to control with statistically significant results by day 34 (P < 0.05) (Figure 5d).

### GLV-1h153-enhanced radiouptake in PANC-1 tumor xenografts and was readily imaged via PET

**Figure 6:**
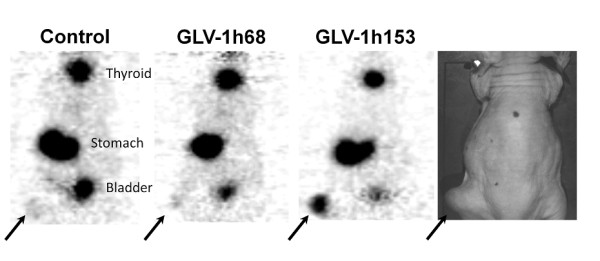
**PET imaging of enhanced radiouptake in GLV-1h153-infected PANC-1 xenografts**. Two × 10^7 ^PFU of GLV-1h153, GLV-1h68, or PBS was injected intratumorally into PANC-1 hindleg tumor-bearing mice. ^124^I-PET scanning was obtained 48 hours after infection and 1 hour after radiotracer administration. GLV-1h153-infected PANC-1 tumors were easily visualized, while no enhanced signal was seen in the PBS- or GLV-1h68 injected tumors. The stomach and thyroid were also imaged due to native NIS expression, and the bladder due to tracer excretion.

After successful cell culture uptake studies we wanted to show the feasibility of using GLV-1h153 in combination with carrier-free ^124^I radiotracer to image infected PANC-1 tumors. hNIS protein expression in the PANC-1 tumor-bearing animals after GLV-1h153 administration was visualized by ^124^I PET. Carrier free ^124^I was IVly administered 48 hours after IT virus injection and PET imaging was performed 1 hour after radiotracer administration. GLV-1h153-injected tumors were easily detected, whereas GLV-1h68- and PBS-injected tumors could not be visualized and therefore were not significantly above background (Figure 6).

## Discussion

Oncolytic viral therapy is emerging as a novel cancer therapy. Preclinical and clinical studies have shown a number of oncolytic viruses to have a broad spectrum of anti-cancer activity and safety [18]. These are ongoing, and the first oncolytic viral therapy has now been approved in China as a treatment for head and neck cancers [19]. Clinical trials are underway to assess the effects of many other oncolytic viral therapies [20]. However, future clinical studies may benefit from the ability to noninvasively and serially identify sites of viral targeting and to measure the level of viral infection and spread in order to provide important information for correlation with safety, efficacy, and toxicity [3-5]. Such real-time tracking would also provide useful information regarding timing of viral dose and administration for optimization of therapy, as well as distribution and replication of the oncolytic virus, and would alleviate the need for multiple and repeated tissue biopsies.

VACV is arguably the most successful biologic therapy agent, since versions of this virus were given to millions of humans during the smallpox eradication campaign [2]. More recently, engineered VACVs have also been successfully used as direct oncolytic agents, capable of preferentially infecting, replicating within, and killing a wide variety of cancer cell types [6-11,13,21]. VACV displays many of the qualities thought necessary for an effective oncolytic antitumor agent. In particular, the large insertional cloning capacity allows for the inclusion of several functional and therapeutic transgenes. With the insertion of reporter genes not expressed in uninfected cells, viruses can be localized and the course of viral therapy monitored in patients.

One such promising virus strain is GLV-1h68[21]. This strain has shown efficacy in the treatment of a wide range of human cancers and is currently being tested in phase I human trials[20]. In this study we describe the generation of a novel recombinant VACV, GLV-1h153, derived from GLV-1h68, which has been engineered for specific targeted treatment of cancer and the additional capability of facilitating noninvasive imaging of tumors and metastases. To our knowledge, GLV-1h153 is the first oncolytic VACV expressing the hNIS protein.

The reporter gene chosen for insertion into GLV-1h153 was based on the already successful PET and SPECT imaging characteristics of the human sodium iodide symporter (hNIS) and carrier-free radioiodine reporter imaging system. hNIS is an intrinsic plasma membrane protein that mediates the active transport and concentration of iodide in thyroid gland cells and some extra-thyroidal tissues [16,17]. Although endogenous NIS is physiologically and functionally expressed in several normal tissues, so far only 2 human cancers - some thyroid cancers, and around 80% of breast cancers including ductal carcinomas - have been shown to express endogenous NIS functionally, making them amenable to radiotherapy [22]. It is one of several human reporter genes that are currently being used in preclinical studies and has even been used in clinical studies imaging prostate cancer [20,23]. hNIS gene transfer via viral vector may allow infected tumor cells to concentrate several commercially available, relatively inexpensive radionuclide probes, such as ^123^I, ^124^I, ^125^I, and ^99m^TcO_4_, all of which have long been approved for human use by the U.S. Food and Drug Administration, allowing noninvasive imaging of tumors expressing NIS [22]. In contrast to a study published by McCart *et al. *[24] using an oncolytic VACV expressing the human somatostatin receptor hSSTR2, hNIS is a transporter-based reporter gene system. Whereas receptors usually have a 1:1 binding relationship with a radiolabeled ligand, transporters provide signal amplification through transport-mediated concentrative intracellular accumulation of substrate. hNIS use has also been shown to be comparable to the commonly used *HSV1-tk *reporter gene [25] and correlated with ^99m^TcO_4 _[26]. This can be very useful for viral distribution with scintigraphy or PET scanning during and after viral therapy, and may allow for correlation with efficacy and toxicity during clinical trials and treatment thus offering potential clinical translation of this dual therapy.

In order to take advantage of the therapeutic and imaging potential of hNIS, several groups have attempted exogenous *NIS *gene transfer in several human cancers including head and neck squamous cell cancers, non-small cell lung, thyroid, liver, colorectal, and prostate cancers, as well as glioma and multiple myeloma [22]. Studies have shown that *hNIS *gene delivery to both thyroidal and non-thyroidal, non-organifying tumor cells is capable of inducing accumulation of therapeutically effective radioiodine doses. For example, a single therapeutic ^131^I dose of 3 mCi was shown to elicit a dramatic therapeutic response in NIS-transfected prostate cell xenografts, with an average volume reduction of more than 90% [27]. Transfection of an *hNIS*-defective follicular thyroid carcinoma cell line with the *hNIS *gene was able to reestablish iodide accumulation activity both in cell culture and in animal models [28]. Furthermore, transfection of pancreatic cancer cells with a replication-deficient adenoviral vector expressing hNIS lead to a more than 15-fold increase in iodide uptake visualized with ^123^I scintigraphy, and an over 75% reduction in volume *in vivo *after treatment with 3mci of ^131^I [29].

We have previously reported on the use of a novel recombinant VACV, GLV-1h99, a derivative of GLV-1h68, which was constructed to carry the human norepinephrine transporter gene (hNET) under the VACV synthetic early promoter placed at the *F14.5L *locus for deep tissue imaging [30,31]. The parental virus GLV-1h68, a recombinant VACV (LIVP strain), was constructed by inserting 3 expression cassettes (*Renilla luciferase*-*Aequorea *green fluorescent protein (RUC-GFP) fusion, β-galactosidase, and β-glucuronidase) into the *F14.5L*, *J2R*, and *A56R *loci of the viral genome, respectively [6]. The hNET protein was expressed at high levels on the membranes of cells infected with GLV-1h99, and expression of the hNET protein did not negatively affect virus replication in cell culture or *in vivo *virotherapeutic efficacy. GLV-1h99-mediated expression of the hNET protein in infected cells resulted in specific uptake of the radiotracer [^131^I]-meta-iodobenzylguanidine ([^131^I]-MIBG). In mice, GLV-1h99-infected tumors, including pancreatic and mesothelioma, were readily imaged by [^124^I]-MIBG PET. However, one of the disadvantages of using hNET is that it requires the carrier MIBG for radioiodine uptake.

GLV-1h153 was effective at infecting and replicating within pancreatic cancer PANC-1 cells as efficiently as its parental virus GLV-1h68. This indicated that insertion of the hNIS protein did not negatively affect virus replication in cell culture. With the hNET-expressing GLV-1h99 virus, on the other hand, there was slight improvement in viral replication and oncolytic effect, which may have been due to the exchange of expression cassettes under the control of promoters with different strengths.

Microarray analysis revealed an almost 2000-fold change increase in hNIS mRNA and an almost 5000-fold change increase by 24 hours after PANC-1 infection with GLV-1h153 at a multiplicity of infection of 5.0. Western blot studies showed hNIS protein expression as a band between 75 and 100 KiloDalton in PANC-1 cells infected with GLV-1h153, with higher concentrations of the protein at higher MOIs. This band also appears in normal human thyroid lysates at a slightly lower molecular weight, which is likely explained by differences in glycosylation within cells [16]. The hNIS protein was successfully transported and inserted into the cell membrane, as demonstrated by fluorescence microscopy. *In vitro*, GLV-1h153-mediated expression of the hNIS protein in infected PANC-1 cells resulted in specific uptake of the radiotracer ^131^I, indicating that the hNIS protein was functional, with the uptake reaching a >70-fold increase compared with uninfected control at an MOI of 1.0.

GLV-1h153 was also effective at infecting, replicating within, and killing PANC-1 cells and eradicating tumor xenografts as efficiently as parental virus GLV-1h68. This indicated that insertion of the hNIS protein did not negatively affect virus replication *in vivo *which was already demonstrated *in vitro*, or the cytolytic activity in cell culture and *in vivo *virotherapeutic efficacy. Similar effects were seen between the IT and IV groups treated with GLV-1h153 or GLV-1h68, indicating the inherent affinity of both genetically modified vaccinia viruses to tumors. Furthermore, administration of GLV-1h153 did not have any significant effects on mean net body weights of the animals 34 days after treatment, with the IT groups even gaining weight as compared to untreated control.

Finally, in mice, three PANC-1 tumors infected with GLV-1h153 were readily detectable by PET, with no enhancement above background of either GLV-1h68- or PBS-treated tumors. Mice were treated intratumorally with GLV-1h153, non-hNIS expressing parent virus GLV-1h68, and PBS, and imaged 48 hours after with carrier free ^124^I. The quantitative ^124^I -PET showed that imaging of GLV-1h153 infection of PANC-1 tumors is feasible after direct tumor injection.

As with any translational therapy, concerns over immune responses remain. Since *hNIS *is a human derived gene, it is unlikely to be immunogenic. However, application of GLV-1h153-mediated hNIS transfer raises concerns over the possibility of autoimmunity in patients. Several papers have already shown that hNIS is not a major candidate for autoimmune disease in patients with patients with Graves' disease and Hashimoto's thyroiditis [32,33]. Moreover, a clinical trial assessing adenoviral-mediated hNIS transfer in humans did not report any serious adverse effects due to autoimmunity in patients treated for prostate cancer [23]. Further studies and caution will be needed to assess the potential of autoimmunity with hNIS transfer in humans. Studies are also now underway to determine the viral biodistribution, as well as the dose and timing related aspects of *in vivo *imaging using this novel virus.

## Conclusions

We have therefore shown that a novel new oncolytic VACV engineered to carry the human sodium iodide symporter gene (*hNIS*) can successfully replicate within and kill pancreatic cancer cells as efficiently as its parental virus GLV-1h68. GLV-1h153 expresses the hNIS reporter gene, which enhances radiouptake *in vitro *and is readily imaged with the clinically approved radiopharmaceutical ^124^I via PET. The ability of GLV-1h153 to infect and enhance cellular uptake of iodine in cells of pancreatic cancer origin, a uniformly fatal disease resistant to conventional therapy, justifies further studies as well as the initiation of clinical trials. Further, this imaging system could be directly translated to human studies, as clinical trials of oncolytic viral therapy would benefit from this noninvasive monitoring modality.

## Abbreviations

ATCC: American Type Culture Collection; (c)DNA: (complementary) deoxyribonucleic acid; CPM: counts per minute; DMEM: Dulbecco's modified Eagle's medium; FDR: false discovery rate; GFP: green fluorescent Protein; gusA: β-glucuronidase; hNIS: human sodium iodide symporter; hNET: human norepinephrin transporter; hSSTR2: human somatostatin receptor; HSV1-tk: herpes simplex virus 1 - thymidine kinase; ^124^I: Iodine-124; ^125^I: Iodine-125; ^131^I: Iodine-131; IT(ly): intratumoral(ly); IV(ly): intravenous(ly); LacZ: β-galactosidase; LDH: lactate dehydrogenase; LIVP: Lister vaccine strain; MIBG: meta-iodobenzylguanidine; MOI: multiplicity of infection; (m)RNA: (messenger) ribonucleic acid; NaClO_4_: sodium perchlorate; PBS: phosphate buffer serum; PCR: polymerase chain reaction; PET: positron emission tomography; ^99m^TcO_4_: 99m-technecium pertechtenate; VACV: vaccinia virus.

## Author disclosure statement

Nanhai G. Chen, Qian Zhang, Yong A. Yu, and Aladar A. Szalay are affiliated with Genelux Corporation. No competing financial interests exist for Dana Haddad, Chun-Hao Chen, Pat Zanzonico, Lorena Gonzalez, Susanne Carpenter, Joshua Carson, Joyce Au, Arjun Mittra, Mithat Gonen, and Yuman Fong.

## Authors' contributions

NC was instrumental in the construction and homologous recombination of GLV-1h153. QZ was instrumental in the hNIS transfer construction and viral sequencing. CC contributed to study design, western blot, and immunofluorescence. YY contributed to the study design and assisted in in vivo studies. SC contributed to the cytotoxicity and flow cytometry assays. JC contributed to the in vitro radiouptake assays. JA contributed to the in vitro radiouptake assays. AM contributed to protein harvesting and western blot. MG was instrumental in statistical analysis of data. PZ was instrumental in study design, imaging experiments, and advice regarding hNIS biology and physiology. YF is the co-corresponding author and was critical to study design and completion. AZ is the corresponding author of this paper and was critical to study design and completion. All authors have read and approved the final manuscript.
